# Alarmin HMGB1 induces systemic and brain inflammatory exacerbation in post-stroke infection rat model

**DOI:** 10.1038/s41419-018-0438-8

**Published:** 2018-03-19

**Authors:** Il-Doo Kim, Hahnbie Lee, Seung-Woo Kim, Hye-Kyung Lee, Juli Choi, Pyung-Lim Han, Ja-Kyeong Lee

**Affiliations:** 1 Department of Anatomy, Inha University School of Medicine, Inchon, Republic of Korea; 2 Medical Research Center, Inha University School of Medicine, Inchon, Republic of Korea; 3 Department of Biomedical Sciences, Inha University School of Medicine, Inchon, Republic of Korea; 4 Department of Brain and Cognitive Sciences, Ewha Womans University, Seoul, Republic of Korea

## Abstract

Post-stroke infection (PSI) is known to worsen functional outcomes of stroke patients and accounts to one-third of stroke-related deaths in hospital. In our previous reports, we demonstrated that massive release of high-mobility group box protein 1 (HMGB1), an endogenous danger signal molecule, is promoted by *N*-methyl-d-aspartic acid-induced acute damage in the postischemic brain, exacerbating neuronal damage by triggering delayed inflammatory processes. Moreover, augmentation of proinflammatory function of lipopolysaccharides (LPS) by HMGB1 via direct interaction has been reported. The aim of this study was to investigate the role of HMGB1 in aggravating inflammation in the PSI by exacerbating the function of LPS. PSI animal model was produced by administrating a low-dose LPS at 24 h post-middle cerebral artery occlusion (MCAO). Profound aggravations of inflammation, deterioration of behavioral outcomes, and infarct expansion were observed in LPS-injected MCAO animals, in which serum HMGB1 surge, especially disulfide type, occurred immediately after LPS administration and aggravated brain and systemic inflammations probably by acting in synergy with LPS. Importantly, blockage of HMGB1 function by delayed administrations of therapeutic peptides known to inhibit HMGB1 (HMGB1 A box, HPep1) or by treatment with LPS after preincubation with HMGB1 A box significantly ameliorated damages observed in the rat PSI model, demonstrating that HMGB1 plays a crucial role. Furthermore, administration of *Rhodobacter sphaeroides* LPS, a selective toll-like receptor 4 antagonist not only failed to exert these effects but blocked the effects of LPS, indicating its TLR4 dependence. Together, these results indicated that alarmin HMGB1 mediates potentiation of LPS function, exacerbating TLR4-dependent systemic and brain inflammation in a rat PSI model and there is a positive-feedback loop between augmentation of LPS function by HMGB1 and subsequent HMGB1 release/serum. Therefore, HMGB1 might be a valuable therapeutic target for preventing post-stroke infection.

## Introduction

Stroke patients are susceptible to infection. In fact, one-third of stroke patient deaths in hospitals can be attributable to post-stroke infection (PSI), especially respiratory and urinary tract infections^1,2^. Numerous mechanisms may underlie increasing risk of PSIs, including the direct consequences of stroke, such as inflammatory activation and brain-induced immune depression, along with indirect factors associated with stroke, such as advanced age and comorbidity^3,4^. Among them, aggravations of brain-specific inflammation and systemic inflammation appear to play critical roles in increasing the frequency of PSI and in worsening functional outcomes of stroke patients^5^.

High-mobility group box 1 (HMGB1), a ubiquitously expressed non-histone DNA-binding nuclear protein, is involved in nucleosome stabilization and gene transcription^6^. However, HMGB1 was reported to act as an endogenous danger-associated molecular pattern (DAMP)^7^ after being released by necrotic cells or actively secreted by macrophages/monocytes into the extracellular environment, thereby triggering and amplifying inflammatory processes^8–10^. Regarding this, relations between HMGB1 plasma levels and disease severity have been reported under various disease conditions such as sepsis^11^, acute pancreatitis^12^, asthma^13^, chronic obstructive pulmonary disease^14^, and stroke^15^. In particular, we and others have reported that in a rat middle cerebral artery occlusion (MCAO) stroke model, massive release of HMGB1 is promoted by *N*-methyl-d-aspartic-induced acute neuronal death, exacerbating neuronal damage and triggering delayed inflammatory processes^16–18^. In addition, we reported that HMGB1 levels in the serum^16^ and cerebrospinal fluid (CSF)^19^ rapidly increased from 3 h post-MCAO, generating dual peaks at 1 day and 6 days post-MCAO (60 min occlusion). Accumulated extracellular HMGB1 mediates acute and delayed damaging processes, especially aggravating inflammation in the postischemic brain^16^.

HMGB1 is known to interact with various molecules, such as lipopolysaccharides (LPS), interleukin-1β (IL-1β), single-stranded DNA, Pam3CSK4, CpG oligodeoxynucleotides, peptidoglycan, and nucleosomes^20–23^, and resulting complexes augment the inflammatory response by binding with receptors of interacting partners in each complex^21–23^. For example, HMGB1 can interact with LPS and transfer it to CD14, thereby enhancing LPS-mediated inflammation^23^. HMGB1 has two LPS-binding domains which bind to polysaccharides or lipid A moieties of LPS, and administration of HMGB1 peptides containing these LPS-binding domains was shown to inhibit LPS-HMGB1 binding, HMGB1-mediated LPS transfer to CD14, and LPS-induced tumor necrosis factor-α (TNF-α) release in human peripheral blood mononuclear cells and in a subclinical endotoxemia mouse model^24^. Therefore, it is possible that accumulated extracellular HMGB1 not only exacerbates secondary damaging process by aggravating inflammation in the postischemic brain but also make animals vulnerable to the following infection.

In the present study, we examined whether or not extracellular HMGB1 accumulated in animals after ischemic insult is involved in induction or aggravation of PSI. LPS administration to animal models of stroke has been used to create PSI models. These administrations involve a single bolus dose of LPS immediately before or after ischemia^25–27^ or low doses of LPS some time after ischemia^28,29^. We used a PSI animal model which had received a single LPS injection at 24 h post-MCAO and confirmed various characteristics of those animals in regards of PSI. We found marked surge of HMGB1 and interaction between HMGB1 and LPS and demonstrated crucial roles of HMGB1 by using peptides known to block interaction between HMGB1 and LPS.

## Results

### HMGB1 levels in the serum and CSF are proportional to severity of ischemic brain damage

**Fig. 1:**
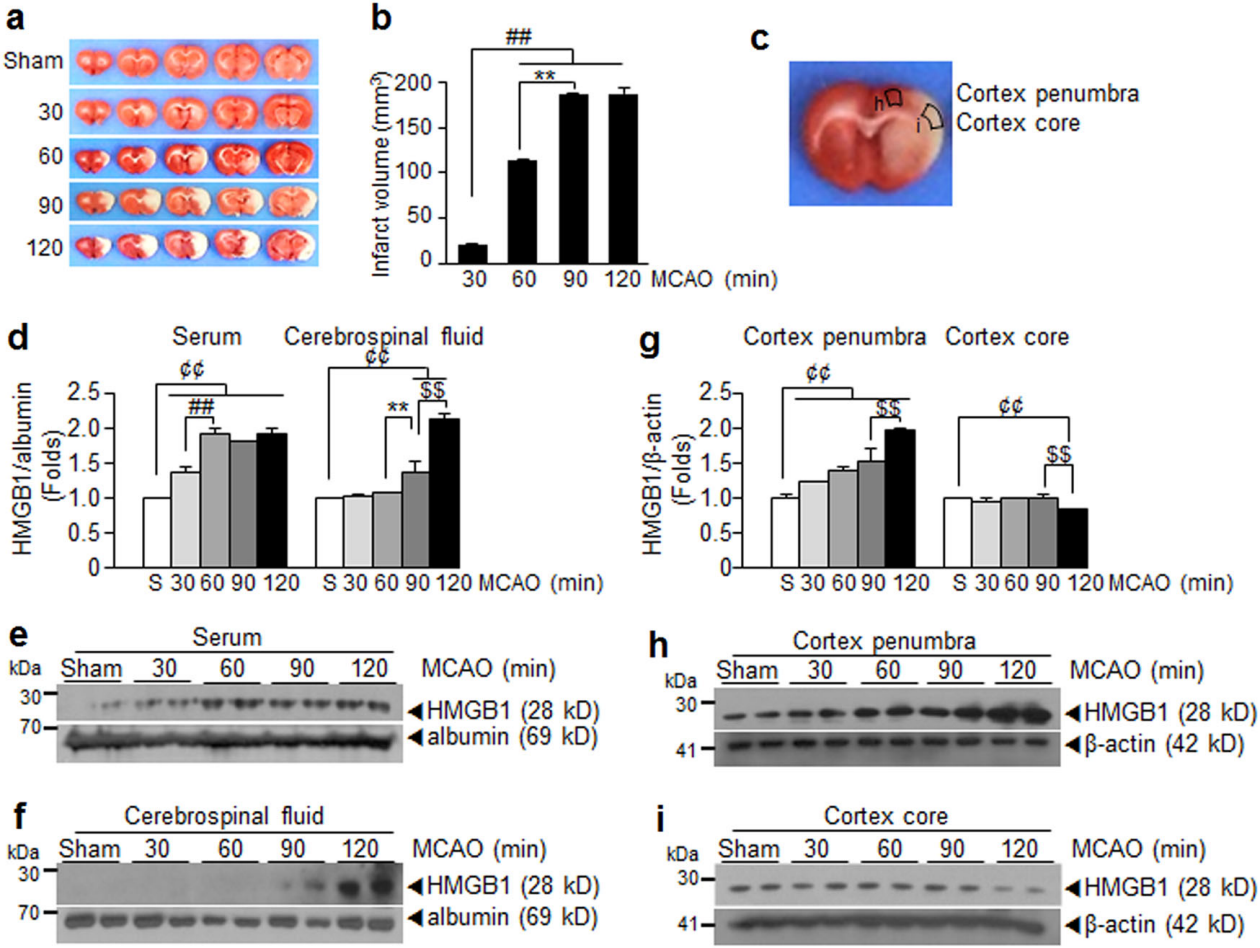
Stroke severity and HMGB1 levels in the serum and CSF. MCA of Sprague–Dawley rats were occluded for 30, 60, 90, or 120 min and mean infarct volumes were assessed at 2 days post-MCAO by TTC staining. Representative images of infarctions in coronal brain sections are presented (**a**) and mean infarction volumes are presented as means ± SEMs (*n* = 6–8) (**b**). Representative images of infarctions in coronal brain sections at 1 day post-MCAO are presented (**c**). Levels of HMGB1 in the serum (**d**, **e**), CSF (**d**, **f**), and in cortex core and penumbra of the ischemic hemisphere indicated in **c** (**g**–**i**) at 1 day post-MCAO (30, 60, 90, or 120 min occlusion) were examined by immunoblotting. Sham or S sham-operated rats. ^¢¢^*p* < 0.01, **p < 0.01, ^$$^*p* < 0.01, ^##^*p* < 0.01 between indicated groups

To investigate the importance of extracellular HMGB1 released after massive neuronal death in the postischemic brain^16,19^, we examined the amounts of HMGB1 released after different durations of ischemic insult. Animals were subjected to MCAO for 30, 60, 90, or 120 min using a suture as previously described (Fig. 1a, b)^19^, and the amounts of HMGB1 in CSF or serum were examined at 1 day post-MCAO. According to the results, the higher the extracellular HMGB1 levels in the serum and CSF, the larger the infarction volumes (Fig. 1d–f), demonstrating a correlation between levels of extracellular HMGB1 and stroke severity in the rat MCAO model. Interestingly, while HMGB1 levels in penumbra increased depending on stroke severity, they decreased in infarct cores due to an acute and massive neuronal cell death in cortices of the ipsilateral hemisphere (Fig. 1c, g–i).

### Systemic LPS administration markedly enhances infarct formation in the postischemic rat brain

**Fig. 2:**
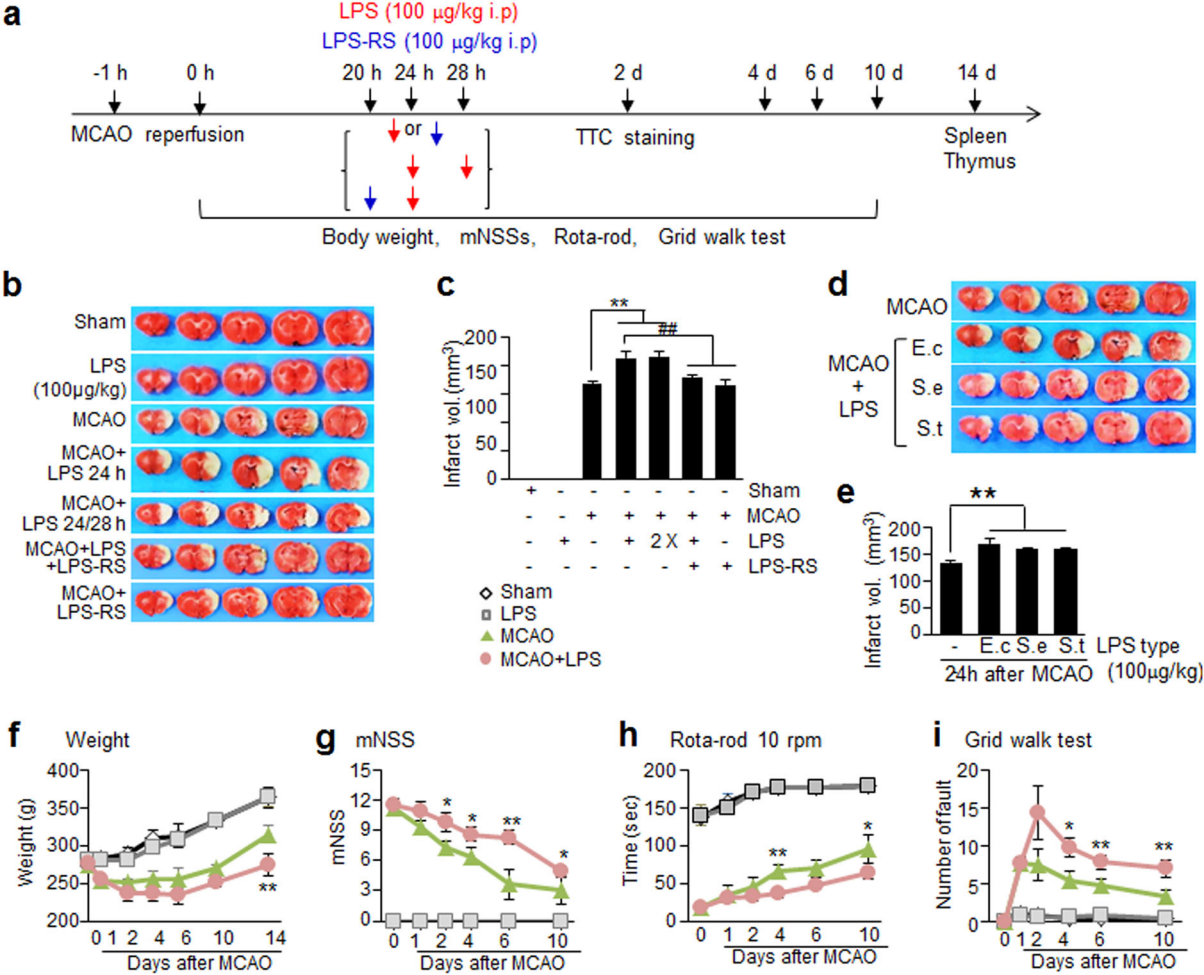
Changes in infarct volumes and weight and aggravation of neurological and motor deficits caused by intraperitoneal injection of LPS in MCAO animals. **a** Each serotype of LPS (100 μg/kg, i.p.) was administered once at 24 h post-MCAO (60 min) or twice (LPS from *E. coli*) at 24 and 28 h post-MCAO and infarct volumes were examined at 2 days post-MCAO. LPS-RS (100 μg/kg, i.p.) was administered once at 24 h post-MCAO or pre-administered 4 h before LPS administration. Representative images of infarctions in coronal brain sections were assessed at 2 days post-MCAO by TTC staining (**b**, **e**) and mean infarction volumes are presented as mean ± SEM (*n* = 3–8) (**c**, **d**). Body weight (**f**) and modified neurological severity scores (**g**) were measured and rotarod test at 10 rpm (**h**) and grid walk test (**i**) were carried out at 1, 2, 4, 6, or 10 days post-MCAO. Results are presented as mean ± SEM (*n* = 3–9). Sham sham-operated rats (*n* = 3), LPS, LPS-treated rats (*n* = 3), MCAO treatment-naive MCAO rats (*n* = 8), MCAO+LPS LPS-treated MCAO rats (*n* = 9), MCAO+LPS-RS LPS-RS-treated MCAO rats (*n* = 3), MCAO+LPS-RS+LPS LPS-RS-treated and LPS-treated MCAO rats (*n* = 4). *p < 0.05, **p < 0.01 vs. treatment-naïve MCAO controls and ^##^*p* < 0.01 vs. LPS-treated MCAO rats

To model PSI, Sprague–Dawley rats were subjected to MCAO for 60 min and 24 h later, LPS was administered systemically (intraperitoneally (i.p.)) (Fig. 2a). Administration of 100 μg/kg of LPS to normal animal had no effect on infarct formation (Fig. 2b, c). However, when the same amount of LPS was administered to MCAO animals, mean infarct volumes measured at 2 days post-MCAO were elevated by 27.1 ± 6.5% (*n* = 7, *p* < 0.01) compared to that in treatment-naïve MCAO controls (Fig. 2b, c), indicating that LPS injection to post-MCAO animals aggravated ischemic damage. In contrast, administration of *Rhodobacter sphaeroides* LPS (LPS-RS), a selective toll-like receptor 4 (TLR4) antagonist^30^, failed to enhance infarct formation in the MCAO+LPS-RS group (Fig. 2b, c). Moreover, enhancement of infarct volume in the MCAO+LPS group was inhibited by co-treating LPS-RS (Fig. 2b, c), indicating that TLR4-dependent signaling was involved in infarct volume expansion. Since administration of LPS twice at 24 and 28 h post-MCAO produced infarct volumes similar to those obtained by single injection at 24 h, we applied a single LPS injection (100 μg/kg, i.p.) at 24 h post-MCAO and used it as an animal model for PSI throughout this study. Comparable infarct expansions were obtained when LPS purified from *Salmonella enterica* or *Salmonella typhimurium* was used (Fig. 2d, e), indicating that increased infarct volume by post-MCAO LPS administration seemed to be a general phenomenon.

### Reduced body weight and exacerbation of functional outcomes in PSI animals

To examine pathophysiological characteristics of PSI animal model, whole body weights, neurological deficits, and motor activities were examined at 1, 2, 4, 6, and 10 days post-MCAO (Fig. 2a). Body weight was significantly lower in the treatment-naïve MCAO control group at 2 days post-MCAO (1 day post-LPS) compared to that of sham controls (Fig. 2f). It was further reduced in the PSI animals (240.3 ± 2.7 g vs. 221.0 ± 3.6 g) and these differences were further elevated at 14 days post-MCAO (287.3 ± 3.6 g vs. 266.3 ± 7.1 g, respectively) (Fig. 2f). In addition, modified neurological severity scores (mNSS) of the PSI animals was significantly higher than that of treatment-naïve MCAO controls at 1 day post-LPS injection and was continuously higher until 10 days post-MCAO (Fig. 2g). Furthermore, the mean time spent on the rotarod at 10 rpm as well as the number of faults in the grid walk test also revealed deterioration of motor functions in PSI animals, which were maintained until 10 days (Fig. 2h,i). These results indicate that post-stroke LPS administration aggravated functional outcomes and these effects were long lasting. Spleen and thymus atrophy observed in the PSI animals (Supplementary Figure S1) further confirmed the establishment of the PSI animal model. Changes in physiological variables were not detected in both normal and MCAO-subjected animals injected with LPS when measured at 24 h post-MCAO or 6 h post-LPS injection (Supplementary Table 1).

### Exacerbation of systemic and brain inflammation in PSI animals

**Fig. 3:**
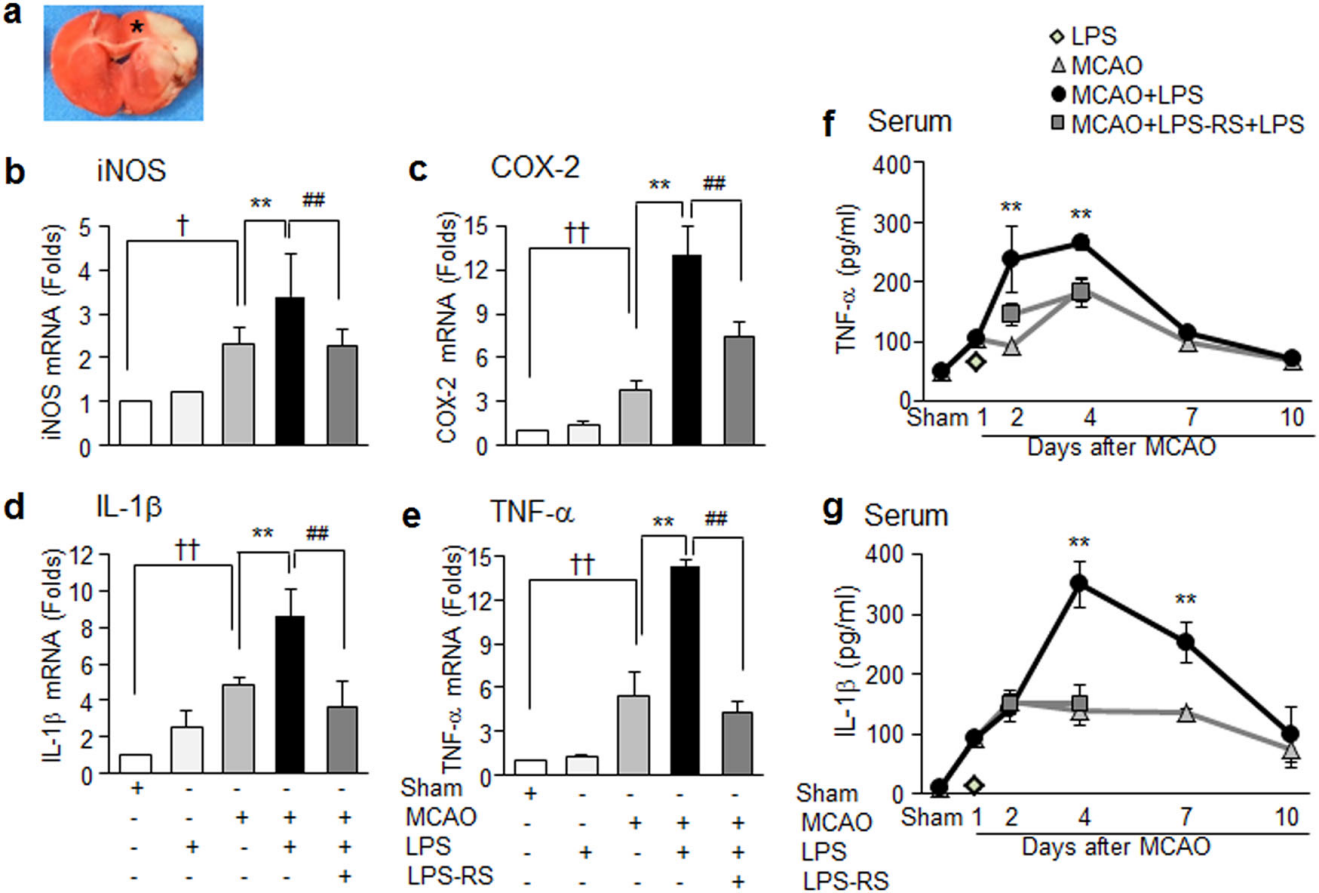
Enhancement of brain and systemic inflammatory marker inductions in PSI animals. Animals were injected with LPS (100 μg/kg, i.p.) at 24 h post-MCAO and levels of inflammatory markers were measured in cortex penumbra of the ischemic hemisphere (**a**) at 36 h post-MCAO by real-time PCR (**b**–**e**) or in the serum at 1, 2, 4, 7, and 10 days post-MCAO by ELISA (**f**,** g**). Results are presented as mean ± SEM (*n* = 4). Sham sham-operated rats, LPS LPS-treated rats, MCAO treatment-naive MCAO rats, MCAO+LPS LPS-treated MCAO rats, MCAO+LPS+LPS-RS LPS-treated and LPS-RS-treated MCAO rats. **p < 0.01 vs. treatment-naive MCAO controls, ^†^*p* < 0.05, ^††^*p* < 0.01, ^##^*p* < 0.01 between indicated groups

Levels of inflammatory markers (inducible nitric oxide synthase (iNOS), TNF-α, cyclooxygenase-2, and IL-1β) in the cortices of ipsilateral hemispheres (Fig. 3a) were significantly elevated in treatment-naïve MCAO controls at 36 h post-MCAO and they were further enhanced in the PSI animals (Fig. 3b–e). In contrast, 100 μg/kg of LPS *per se* was unable to induce inflammatory markers except for moderate induction of IL-1β. In addition, administration of LPS-RS to PSI animals inhibited further enhancement of inflammatory marker inductions observed in PSI animals (Fig. 3b–e). Moreover, we found that circulating TNF-α and IL-1β levels in the serum were also significantly enhanced in the PSI animals compared to those in treatment-naïve MCAO controls at 2, 4, or 7 days post-MCAO with different kinetics, that is, earlier enhancement of TNF-α and delayed and sustained enhancement of IL-1β (Fig. 3f, g). Differences in both cytokine levels in the serum were not detected at 10 days post-MCAO (Fig. 3f, g). We found significant enhancement of inflammatory cytokine levels in the contralateral side brains of PSI animals, although they were not changed in the MCAO animals (Supplementary Figure S2). However, as expected, such enhancement of serum TNF-α and IL-1β levels were not detected in the LPS-RS-administered PSI animals (Fig. 3f, g). Results indicate that systemic inflammatory challenge after MCAO exacerbated both systemic and brain inflammation for almost 10 days and it was dependent on TLR4 signaling.

### Enhanced accumulation of disulfide-type HMGB1 in the serum of PSI animals

**Fig. 4:**
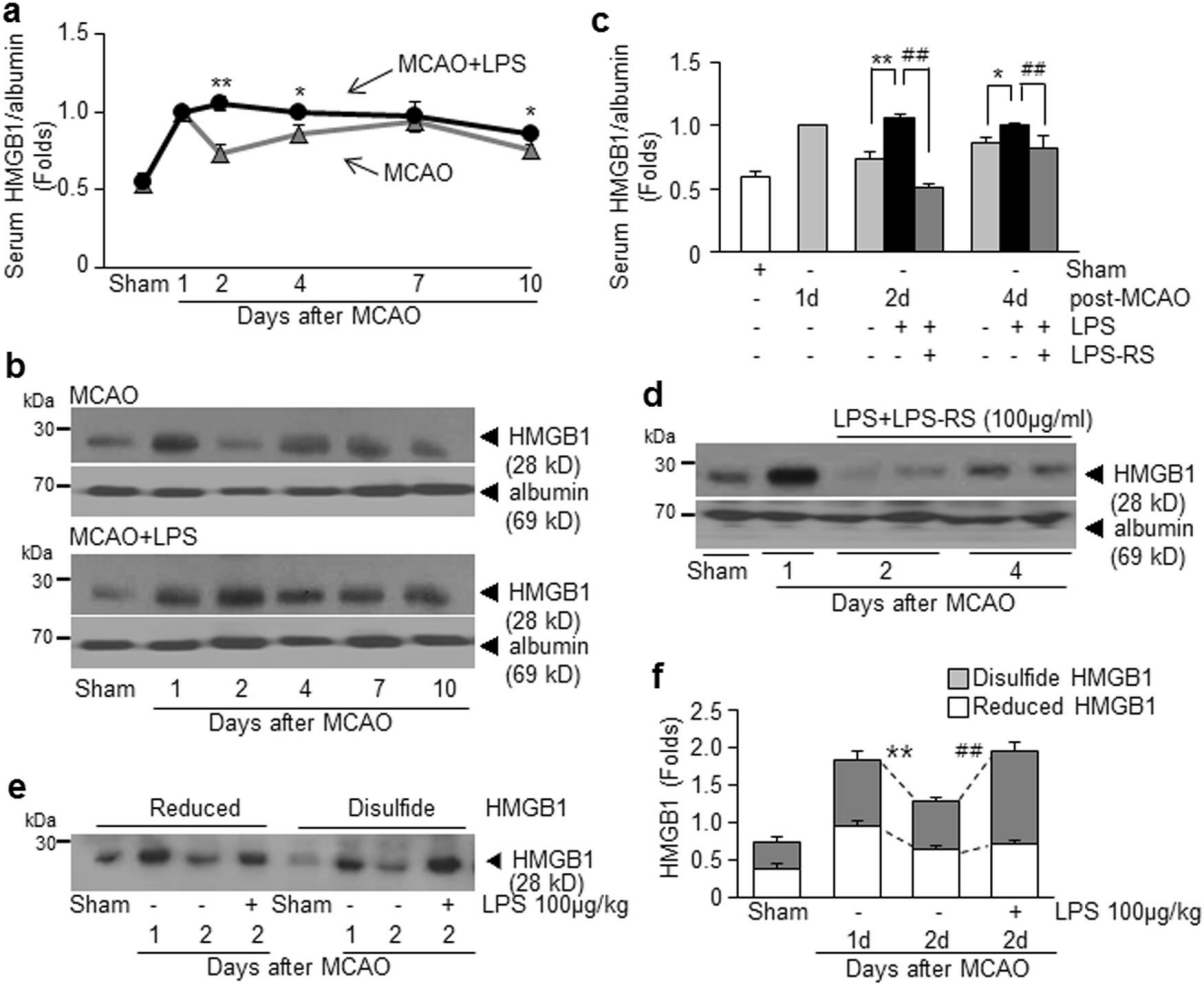
HMGB1 surge in the serum of PSI animals. Serum HMGB1 levels were examined for the MCAO or PSI (100 μg/kg of LPS injected at 24 h post-MCAO) animals at 1, 2, 4, 7, and 10 days post-MCAO (**a**,** b**) or for the MCAO+LPS+LPS-RS group (**c**, **d**) at 2 and 4 days post-MCAO by immunoblotting. Consecutive pull-down assay using biotinylated-iodoacetamide (IAM) and biotinylated-maleimide (M) was carried out with sera obtained at 1 or 2 days post-MCAO in the presence or absence of LPS treatment (Supplementary Figure 2). **e**, **f** Relative levels of disulfide-HMGB1 or reduced-HMGB1 were examined four independent experiments and representative pictures are presented (**e**) and results are presented as the mean ± SEM (**f**). Sham sham-operated rats, MCAO treatment-naive MCAO rats (*n* = 5), MCAO+LPS LPS-treated MCAO rats (*n* = 5), MCAO+LPS+LPS-RS LPS-treated and LPS-RS-treated MCAO rats (*n* = 4). *p < 0.05, **p < 0.01 vs. treatment-naive MCAO controls, ^##^*p* < 0.01 vs. LPS-treated MCAO group

To examine levels of systemic HMGB1 under PSI conditions, serum HMGB1 levels were examined at 1, 2, 4, 6, or 10 days post-MCAO (1, 3, 5, or 9 days post-LPS injection, respectively). In treatment-naïve MCAO controls, serum HMGB1 levels surged with dual peaks at 1 day and 7 days post-MCAO (Fig. 4a, b), which was in accordance with our previous findings^16,19^. Interestingly, in the PSI animals, serum HMGB1 level increased further at 2 days post-MCAO instead of declining and such enhanced HMGB1 levels were maintained until 10 days post-MCAO (Fig. 4a, b). Moreover, serum HMGB1 up-regulation was not observed in the LPS-RS-administered PSI animals (Fig. 4c, d). These results indicate that HMGB1 release/secretion into the extracellular space was markedly up-regulated after LPS injection, which might further exacerbate brain and systemic inflammation via a vicious positive-feedback cycle, in which TLR4 plays an important role. To investigate the oxidative status of HMGB1 accumulated in sera of PSI animals, pull-down assay was carried out using iodoacetamide (IAM) and maleimide (M), known to bind to free thiol groups (Supplementary Figure S3). Serum was prepared at 1 or 2 days post-MCAO with or without LPS administration and subjected to consecutive pull-down assay after incubation with biotinylated-IAM (biotin-IAM) and then with biotinylated-M (biotin-M). Results revealed that HMGB1 accumulated in sera of treatment-naïve MCAO controls at 1 or 2 days post-MCAO was a mixture of reduced and disulfide forms (Fig. 4e, f). Interestingly, only disulfide form was significantly increased in sera of the PSI animals at 2 days post-MCAO (Fig. 4e, f). Since disulfide-HMGB1 is known to interact with LPS^31^, significant increase in disulfide-HMGB1 in the PSI animals might underlie the inflammatory exacerbation observed in this group.

### HMGB1 antagonistic peptide mitigates exacerbation of systemic inflammation in PSI animals

**Fig. 5:**
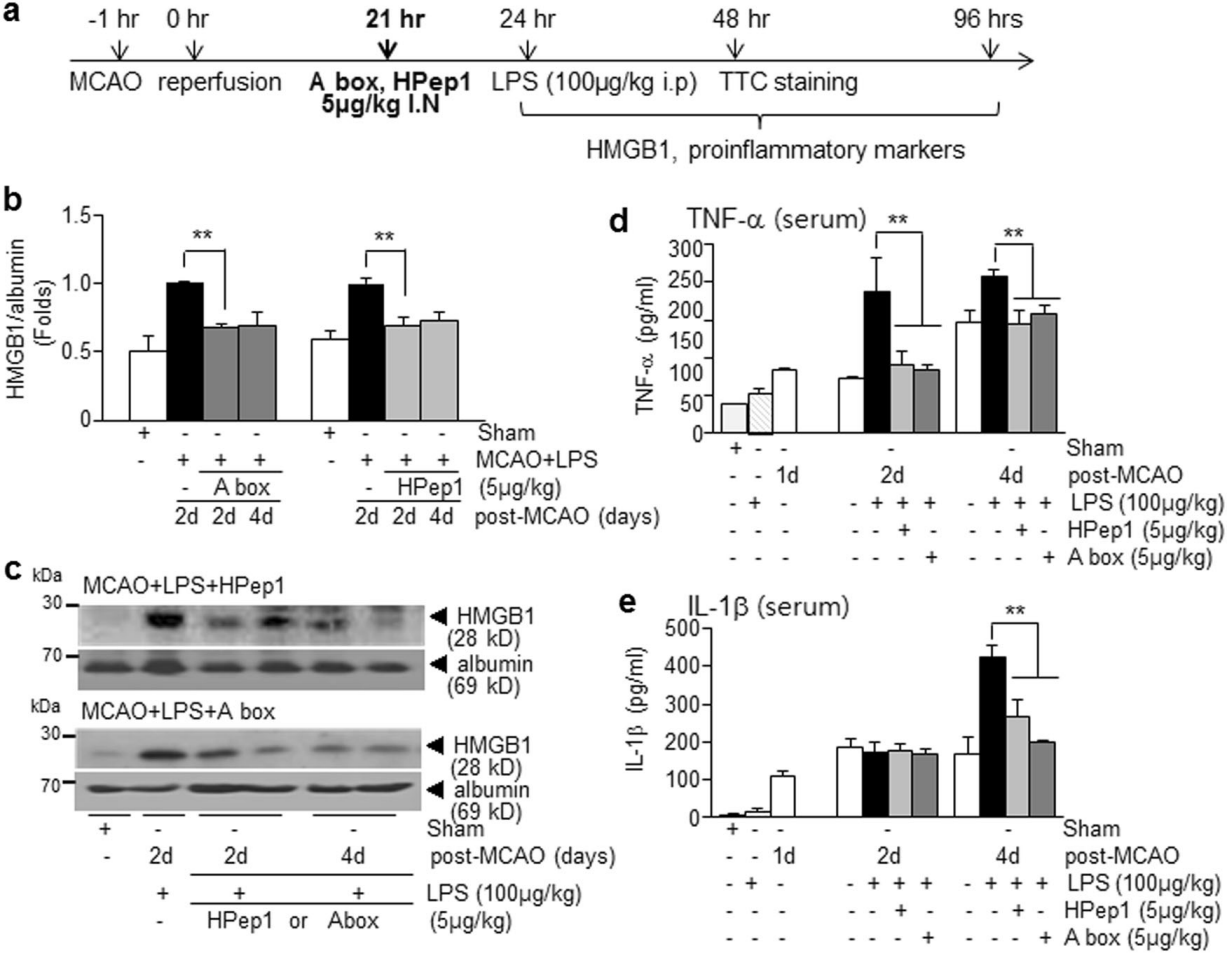
Suppression of systemic inflammatory aggravation in PSI animals by blocking HMGB1 function. **a** Animals were administered HMGB1 A box (5 μg/kg) or HPep1 (5 μg/kg) intranasally 3 h prior to LPS administration (100 μg/kg, i.p.) at 24 h post-MCAO. **b**, **c** Serum HMGB1 levels were examined at 2 or 4 days post-MCAO for MCAO or PSI animals with or without A box or HPep1 treatment at 21 h post-MCAO by immunoblotting. **d**, **e** Levels of TNF-α and IL-1β in the serum were measured in MCAO, MCAO+LPS, MCAO+LPS+A box (5 μg/kg), or MCAO+LPS+HPep1 (5 μg/kg) group at 2 days post-MCAO by ELISA. Results are presented as means ± SEMs (*n* = 4). Sham or S sham-operated rats, LPS LPS-treated rats, MCAO treatment-naive MCAO rats, MCAO+LPS LPS-treated MCAO rats, MCAO+LPS+A box A box-treated MCAO+LPS rats, MCAO+LPS+HPep1 HPep1-treated MCAO+LPS rats. **p < 0.01 vs. LPS-treated MCAO group

To determine whether HMGB1 plays an important role in the aggravations of inflammation and brain damage in the PSI animal, we inhibited HMGB1 function using HMGB1 A box, a competitive antagonistic peptide of HMGB1^32^, or HPep1, HMGB1 peptide known to inhibit LPS binding to HMGB1 or LPS-binding protein^24^. When HMGB1 A box was administered intranasally at 21 h post-MCAO (3 h prior to LPS injection) (Fig. 5a), HMGB1 up-regulation in the serum of PSI animals observed at 2 days after LPS administration (1 day post-LPS administration) (Fig. 4a, b) was significantly suppressed (Fig. 5b, c). Similarly, serum HMGB1 up-regulation at 4 days after LPS administration (3 days post-LPS administration) was also significantly suppressed (Fig. 5b, c). Comparable levels of suppression were detected after administering HPep1 at 21 h post-MCAO (Fig. 5b, c). In addition, serum TNF-α and IL-1β up-regulation observed in PSI animals at 2 or 4 days post-MCAO (Fig. 3f, g) was completely suppressed by A box or HPep1 injections at 3 h prior to LPS administration (Fig. 5d, e). These results indicate that delayed suppression of HMGB1 function by antagonistic peptides mitigated LPS-induced systemic inflammation.

### HMGB1 antagonistic peptide ameliorates exacerbation of brain inflammation and reduces infarct volume expansion in PSI animals

**Fig. 6:**
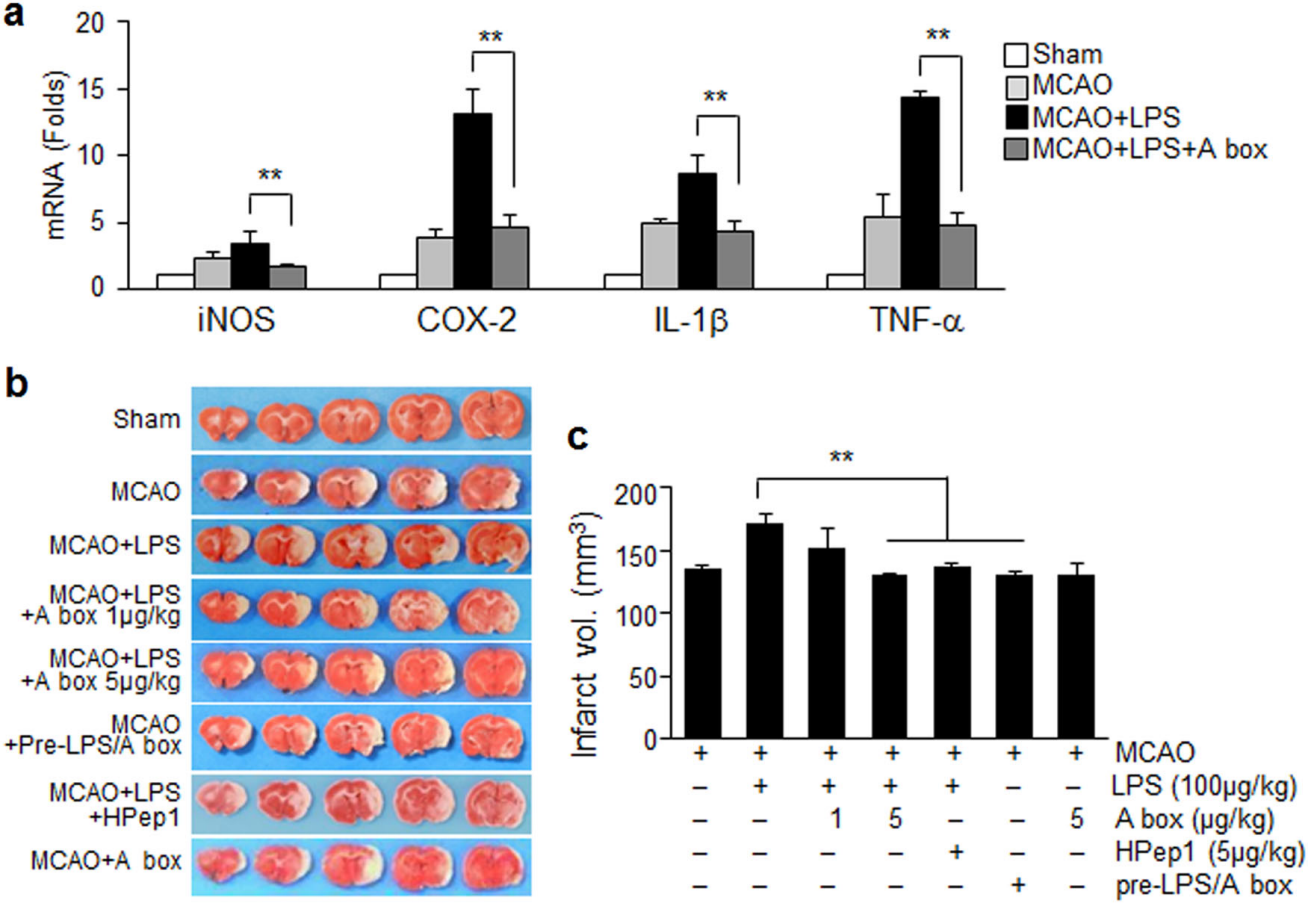
Suppression of brain inflammatory aggravation and infarct expansion in PSI animals by blocking HMGB1 function. HMGB1 A box (5 μg/kg) or HPep1 (5 μg/kg) was administered intranasally 3 h prior to LPS administration (100 μg/kg, i.p.) at 24 h post-MCAO or LPS was preincubated with HMGB1 A box for 6 h before administration to MCAO as shown in Fig. 5a.**a** Levels of inflammatory markers in cortex penumbra of the ischemic hemisphere (Fig. 3a) were measured at 2 days post-MCAO by immunoblotting. Results are presented as mean ± SEM (*n* = 4). **b**, **c** Representative images of infarctions in coronal brain sections obtained were assessed at 2 days post-MCAO by TTC staining (**b**) and mean infarction volumes are presented as mean ± SEM (*n* = 6–8) (**c**). Sham sham-operated rats (*n* = 3), MCAO treatment-naive MCAO rats (*n* = 8), MCAO+LPS LPS-treated MCAO rats (*n* = 9), MCAO+LPS+A box A box-treated MCAO+LPS rats (*n* = 7), MCAO+LPS+HPep1 HPep1-treated MCAO+LPS rats (*n* = 4), MCAO+LPS+Pre-A box MCAO+LPS rats treated with LPS after preincubation with A box (*n* = 3). **p < 0.01 vs. LPS-treated MCAO group

Intranasal administration of HMGB1 A box (5 μg/kg) at 21 h post-MCAO significantly suppressed enhancement of inflammatory marker induction in brain parenchyma (cortical penumbra; Fig. 3a) observed in the PSI animals at 2 days post-MCAO (Fig. 6a). Expansion of infarct volumes observed in the PSI animals at 2 days post-MCAO was not detected after administering HMGB1 A box (5 μg/kg) or HPep1 (5 μg/kg) (Fig. 6b, c). However, when LPS was preincubated with HMGB1 A box for 6 h before injection, LPS failed to induce infarct volume expansion (Fig. 6b, c). These results indicate that HMGB1 plays a critical role in aggravation of systemic and brain inflammation associated with PSI; therefore; delayed suppression of HMGB1 function mitigated not only inflammatory aggravation but also infarct volume expansion in the brain of PSI animals. No changes in physiological parameters were detected in the HMGB1 A box-treated PSI animals (Supplementary Table 1).

### Direct binding between HMGB1 and LPS in sera of PSI animals

**Fig. 7:**
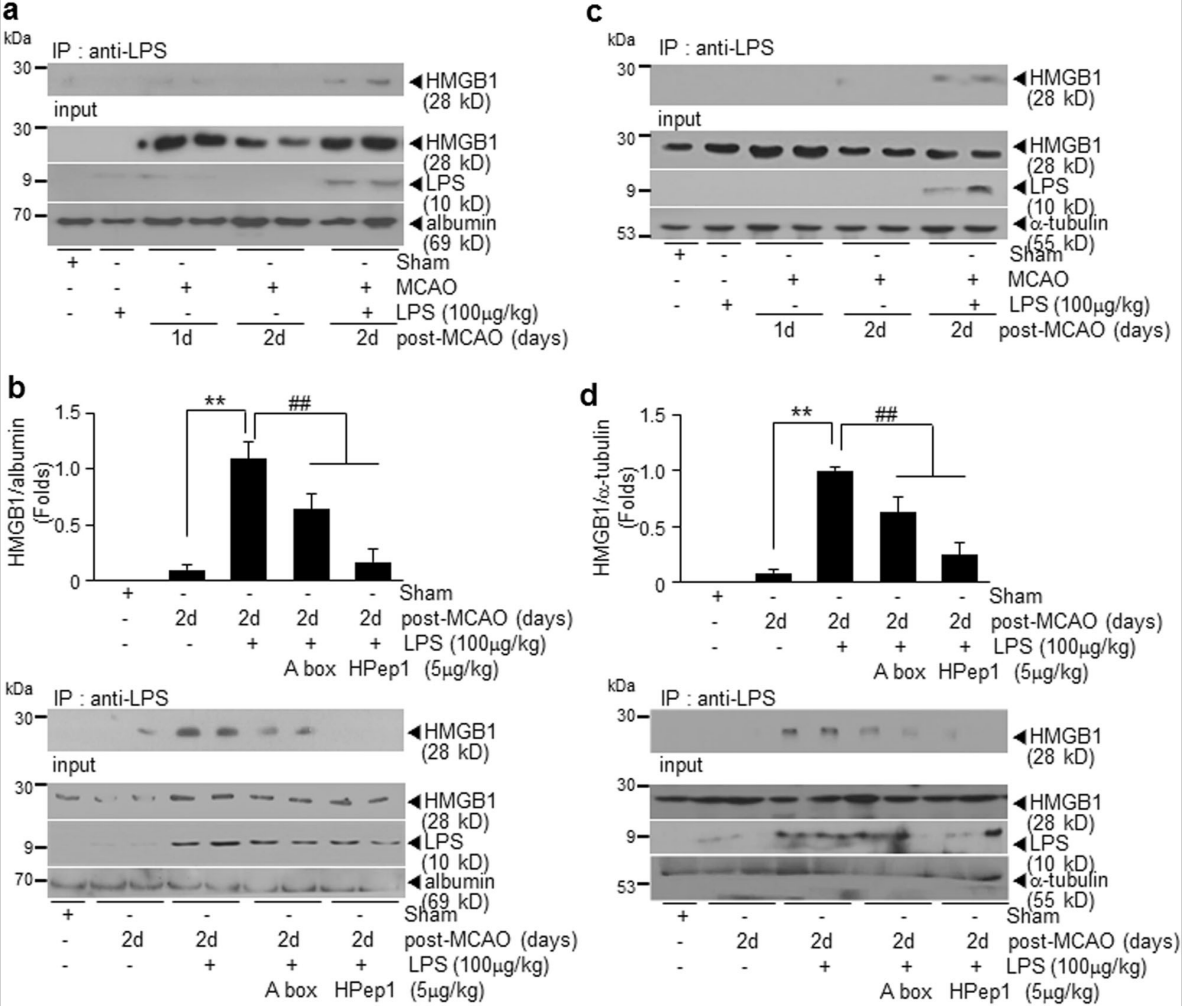
Enhanced binding between LPS and HMGB1 in sera of LPS-injected MCAO animals and inhibition of LPS-HMGB1 binding by blocking HMGB1. Binding between HMGB1 and LPS was examined by co-immunoprecipitation-immunoblotting assay. **a** Serum was prepared from MCAO or PSI animals at 1 or 2 days post-MCAO and precipitated with anti-LPS antibody, and amounts of HMGB1 were determined by immunoblotting using anti-HMGB1 antibody. Amounts of HMGB1, LPS, or serum albumin before immunoprecipitations are presented as input controls. **b** Serum was prepared from MCAO or PSI animals at 2 days post-MCAO after pre-administrating HMGB1 A box (5 μg/kg) or HPep1 (5 μg/kg) at 21 h post-MCAO, and the same co-immunoprecipitation experiments were carried out. **c**, **d** Co-immunoprecipitation-immunoblotting assay was carried out using brain tissues prepared from the cortical penumbra of MCAO or PSI animals at 2 days post-MCAO. Results are presented as mean ± SEM (*n* = 4). Sham sham-operated rats. **p < 0.01 vs. treatment-naive MCAO controls, ^##^*p* < 0.01 vs. LPS-treated MCAO group

To demonstrate direct binding between HMGB1 and LPS, co-immunoprecipitation assays were carried out with sera and brain tissues prepared from PSI animals. HMGB1–LPS complex was detected in sera of PSI animals prepared at 1 day after LPS treatment (2 days post-MCAO) but not in the one prepared from treatment-naive MCAO control (Fig. 7a). HMGB1–LPS complex was significantly decreased or completely disappeared when serum proteins were prepared from animals administered HMGB1 A box (5 μg/kg) or HPep1 (5 μg/kg), respectively, at 21 h post-MCAO (Fig. 7b), indicating that HMGB1 present in the serum of MCAO animals formed a complex with LPS. The HMGB1–LPS interaction was also detected in brain tissues prepared from cortices of PSI animals at 1 day after LPS treatment, although the amount of HMGB1–LPS complex was low, but these complexes were significantly decreased in HMGB1 A box-treated or HPep1-treated PSI animals (Fig. 7c, d). Binding activity of HMGB1 accumulated in sera of LPS-injected MCAO animals to LPS was further confirmed in in vitro pull-down assay (Supplementary Figure S4). These results demonstrate that HMGB1 accumulated in the brain and serum of ischemic animals interacted with systemically administered LPS.

## Discussion

Our aim was to investigate the role of HMGB1 in the initiation and aggravation of PSI in a rat stroke model. We detected profound aggravations of both brain and systemic inflammation and behavioral outcomes in our PSI animal model, in which HMGB1 release/secretion was markedly up-regulated immediately after LPS administration and mediated aggravation of the above-mentioned symptoms by acting in synergy with LPS (Supplementary Figure S5). Although numerous reports have shown the impact of PSI on the functional outcomes of stroke patients, this is the first report presenting a DAMP molecule, HMGB1, as a possible endogenous mediator.

In the present study, we found various inflammatory markers were significantly up-regulated in a stroke animal model (MCAO) and further elevated by LPS injection in both brain parenchyma and serum (Fig. 3). Aggravation of neurological outcomes, infarct volume expansion (Fig. 2), and spleen and thymus atrophy observed in the PSI animals (Supplementary Figure S1) further confirmed the establishment of the PSI animal model. Weight reductions of thymus and spleen were detected at 6 days post-MCAO, more delayed time points, which was in agreement with previous reports on early activation and delayed severe immunodepression in the peripheral immune system of the postischemic animal^33^. These changes were rapidly aggravated by delayed systemic inflammation occurring under PSI, resulting in sustained inflammation and causing devastating effects on ischemic outcome. Considering that administration of the same amount of LPS (100 μg/kg) into normal animals had almost no effect on cytokine levels in both brain parenchyma and serum and that administration of LPS-RS failed to induce all these aggravation symptoms, the effects of LPS were potentiated in animals after ischemic insult, and TLR4 signaling was involved in this potentiation. In addition, marked suppression of these symptoms by administration of HMGB1 A box or HPep1 or by LPS treatment after pre-incubating it with HMGB1 A box (Figs. 5 and 6) indicates that HMGB1 might play a critical role in promoting LPS functions, thereby aggravating brain and systemic proinflammatory processes and damage to brain-modulated functions in LPS-administered MCAO animals.

In a previous study, HMGB1 was shown to rapidly accumulate in the serum^16^ and CSF^19^ of a MCAO animals (60 min) starting from 3 h post-MCAO, generating dual peaks at 1 day and 6–7 days post-MCAO, respectively. In terms of the origin of HMGB1 released in the blood stream, it is from necrotic cells in ischemic brain^16,18,19^ and also from other cell types, such as activated leukocytes^34^. In the present study, the serum HMGB1 level was not reduced at 2 days post-MCAO (1 day post-LPS), but instead further elevated in LPS-administered MCAO animals, and this up-regulation was maintained until 10 days post-MCAO (9 days post-LPS) (Fig. 4). This result suggests a positive-feedback loop between augmentation of exogenous LPS function by accumulated HMGB1 at 1 day post-MCAO and up-regulation of HMGB1. Therefore, LPS injection with a single bolus (100 μg/kg) at 24 h post-MCAO triggered this vicious cycle, which lasted for more than 10 days (Fig. 4). Delayed treatment with HMGB1 A box or HPep1 effectively suppressed HMGB1 up-regulation in the serum at 2 or 4 days post-MCAO (1 or 3 days post-LPS) (Fig. 5b, c), which was coincided with suppression of all deterioration symptoms observed in the PSI animal model (Figs. 5 and 6, Supplementary Figure S1). In the present study, we demonstrated the association between plasma HMGB1 levels and disease severity in an animal model of MCAO (Fig. 1), which has been documented in stroke patients^34^ and might explain the vulnerability of severe stroke patients to upcoming PSI^2^.

Accumulating evidence indicates that not only the amount but the oxidation status of released HMGB1 plays critical roles in determination of pathological conditions^35–38^. We observed that both disulfide-HMGB1 and reduced-HMGB1 were in the serum of the MCAO group at 1 or 2 day post-MCAO, in contrast, only the disulfide form was elevated in the PSI animals at 1 day after LPS injection (Fig. 4e, f). The presence of both disulfide-HMGB1 and reduced-HMGB1 in the serum of stroke patients and stroke-induced mice at 24 h post-MCAO (90 min) has been previously observed^39^, which is in agreement with our report (Fig. 4e, f). However, it has been reported that the disulfide-HMGB1 is critical for priming microglia to exacerbate the neuroinflammatory response upon exposure to subsequent inflammatory stimuli, such as LPS, ex vivo^40,41^, and it is engaged with TLR4 to conduct those functions^31^. Importance of TLR4-dependent signaling has been reported in inflammatory aggravation after acute stressor and subsequent LPS treatment^42^, which also corroborates our results, in which inflammatory aggravation was suppressed in the LPS-RS-treated PSI animal model (Figs. 2 and 3). Since the number of fatty acids is the major determinant of the immunogenicity of endotoxin, LPS-RS containing underacylated lipid A structures (four to five fatty acids) is markedly less effective and can inhibit the strong endotoxic response triggered by hexa-acylated LPS by competitive binding to TLR4^43^. In the present study, direct binding between LPS and HMGB1 was detected in the serum at 1 day post-LPS administration (Fig. 7) and the potency of serum HMGB1 binding to LPS was further confirmed in pull-down assay using size-fractionated serum proteins and biotinylated-LPS (biotin-LPS) (Supplementary Figure S4). Recently, it has been reported that in addition to LPS, peptidoglycan and HMGB1 synergistically induces iNOS expression and the peptidoglycan/rHMGB1 complex increases phosphorylation of JNK and p38 as well as activates nuclear factor-κB through receptor for glycation end product (RAGE) and TLR2 ligands^20^, further supporting the possibility that HMGB1 may function as a mediator of inflammatory aggravation in PSI.

A key finding of this study is the profound effect of systemic inflammatory challenge on brain inflammation and brain-modulated functional outcomes, which took place in conjunction with significant expansion of infarct volume (Fig. 2). Although, there is a controversy regarding infarct volume increase in PSI animals^28,29^, we observed a significant increase in infarction volume at 2 days post-MCAO, when LPS was administered 24 h post-MCAO (Fig. 2). Furthermore, TLR4 plays a critical role in all these processes, as LPS-RS failed to induce them (Fig. 2–4). We were able to detect direct binding between LPS and HMGB1 in brain parenchyma at 1 day post-LPS administration, albeit relatively weak band intensity due to the extremely small amount of LPS (Fig. 7c, d).

In the present study, LPS-induced inflammatory aggravation occurred very rapidly both in the serum and brain parenchyma of MCAO animals, at 1 day after LPS treatment (Fig. 3), resulting in prolonged inflammation and devastating functional outcomes in PSI. However, in addition to aggravation of acute inflammation, clinical studies suggest that stress-mediated immunodepression driven by the sympathetic nervous system (SNS) and hypothalamo-pituitary-adrenal (HPA) axis also serves as an essential facilitating factor in the onset of PSI^44,45^ Regarding this, it has been recently reported that RAGE, a well-known HMGB1 receptor^46^, mediates inactivation of nicotinic acetylcholine receptors in sympathetic neurons under high glucose conditions^47^. Furthermore, HMGB1-RAGE signaling is involved in functional exhaustion of mature monocytes and lymphopenia after ischemic injury, which is a hallmark of immunodepression after ischemia, thereby affecting the peripheral immune response^39^. Thus, further investigation is required on whether or not massive release of HMGB1 is involved in activation of the HPA axis and SNS or immunodepression after cerebral ischemia, thereby increasing vulnerability to infection.

In addition to cerebral ischemia, this mediator-like function of HMGB1 with respect to induction of delayed systemic inflammatory challenge might be relevant under various neuropathological conditions, such as epilepsy or spinal cord injury, as they feature massive serum HMGB1 accumulation due to acute and massive neuronal death^48^. Therefore, modulation of HMGB1 might serve as a therapeutic target for preventing PSI and further studies are needed to explore this possibility.

## Materials and methods

### Surgical procedure used for MCAO

Male Sprague–Dawley rats were housed under diurnal lighting conditions and were allowed access to food and tap water ad libitum. All animal studies were carried out in strict accordance with the Guide for the Care and Use of Laboratory Animals published by the National Institute of Health (NIH, USA 2013) and performed in accordance with ARRIVE guidelines (http://www.nc3rs.org/ARRIVE). The animal protocol used in this study was reviewed and approved by the INHA University-Institutional Animal Care and Use Committee (INHA-IACUC) with respect to ethicality (Approval Number INHA-141124-337). MCAO was carried out as previously described^19^. In brief, male Sprague–Dawley rats (250–300 g) were anesthetized with 5% isoflurane in 30% oxygen/70% nitrous oxide and maintained during surgery using 0.5% isoflurane in the same gas mixture. Occlusion of the right middle carotid artery was induced for 1 h by advancing a nylon suture (4-0; AILEE, Busan, South Korea) with a heat-induced bulb at its tip (∼0.3 mm in diameter) along the internal carotid artery for 20–22 mm from the bifurcation of the external carotid artery. Reperfusion was allowed for up to 2 days. A thermoregulated heating pad and heating lamp were used to maintain a rectal temperature of 37 ± 0.5 °C during surgery. Animals were randomly allocated to sham (*n* = 12), MCAO (*n* = 38), LPS-treated (*n* = 10), MCAO+LPS-treated (24 h post-MCAO) (*n* = 28), MCAO+LPS-treated (24/28 h post-MCAO) (*n* = 4) groups, MCAO+LPS+A box-treated (*n* = 11), MCAO+LPS+HPep1-treated (*n* = 14), MCAO+A box-treated groups (*n* = 3), MCAO+LPS-RS-treated (*n* = 3), and MCAO+LPS+LPS-RS-treated groups (*n* = 8). Animals allocated to the sham group underwent an identical procedure but the MCA was not occluded. In general, mortality was not observed during surgery, but mortality after surgery was 6.9% (9/130).

### Treatment with LPS or LPS-RS

LPS (from *Escherichia coli* (E. coli), *Salmonella enterica* (SE), *Salmonella typhosa* (ST)) (100 μg/kg; Sigma Aldrich, St. Louis, MO, USA; L6529, L7770, L6143) was administered i.p. at 24 or 28 h post-MCAO. Similarly, LPS-RS (LPS from *Rhodobacter sphaeroides*; TLR4 antagonist) (Invivogen, San Diego, CA, USA; tlrl-rslps) was administered intraperitoneally at 24 h post-MCAO. Solution concentrations were adjusted to allow an injected volume of 0.3 ml in PBS. For the preincubation experiment, LPS (100 μg/kg) was preincubated with 5 μg/kg of HMGB1 A box (HMGbiotech, Milano, Italy; HM-012) and then administered intraperitoneally.

### Intranasal administration

Intranasal administrations were carried out as previously described by Kim et al^49^. Briefly, at 21 h post-MCAO, rats were anesthetized with an intramuscular injection of a mixture of ketamine (3.75 mg/100 g body weight) and xylazine hydrochloride (0.5 mg/100 g body weight). A nose drop containing 1 or 5 μg/kg of HMGB1 A box or HPep1 in PBS (20 μl) was carefully placed in each nostril of anesthetized animals (supine 90° angle) using a sterile yellow tip. The procedure was repeated until the entire dosage had been administered with 2-min intervals between applications.

### Infarct volume assessment

Coronally sectioned (2 mm) brain slices were immediately stained with 2% 2,3,5-triphenyl tetrazolium chloride (TTC) (37 °C for 15 min) and fixed in 4% paraformaldehyde. Infarcted tissue areas were measured using the Scion Image program (Scion Corporation, Frederick, MD, USA). To correct for brain edema following ischemia, measured infarct areas were adjusted with respect to areas in contralateral hemispheres. Infarct volumes were calculated (in mm^3^) by summing infarct sizes on adjacent tissue sections.

### Modified neurological severity scores

Neurological deficits were evaluated using mNSS at 2 days post-MCAO. The mNSS system is based on the results of four tests, namely, motor, sensory, balance, and reflex tests, all of which are graded using a 0 to 18 scale (normal: 0; maximal deficit: 18)^50^. Motor scores were determined by: (1) suspending a rat by its tail and awarding a score of zero or one for each of the following (total score 0–3); forelimb flexion, hindlimb flexion, head movement by >10° with respect to the vertical axis within 30 s; and by (2) placing a rat on the floor and awarding scores from 0 to 3 for each of the following: normal walking, 0; inability to walk straight, 1; circling toward the paretic side, 2; or falling on the paretic side, 3. Sensory tests included a placing test (score 0–1) and a proprioceptive test (score 0–1). The beam balance test was used to test balance and scores from 0 to 6 were allocated as follows: balancing with a steady posture, 0; grasping the side of the beam, 1; hugging the beam with one limb off the beam, 2; hugging the beam with two limbs off the beam or rotating around the beam for over 60 s, 3; attempting to balance on the beam but falling off within 20 to 40 s, 4; attempting to balance on the beam but falling off within 20 s, 5; or making no attempt to balance or hang onto the beam, 6. Reflex test scores were determined by awarding scores to the following four items (maximum possible score of 4): pinna reflex, 0–1; corneal reflex, 0–1; startle reflex, 0–1; seizures, myoclonus or myodystony, 0–1.

### Rotarod test

At 24 h before MCAO, rats were conditioned on a rotarod unit at a constant 3 rpm until they were able to remain on the rotating spindle for 180 s. One day after MCAO, each rat was subjected to a test trial on the rotarod at 10 rpm, and residence times on the rotarod were measured at 1, 2, 4, 6, and 10 days post-MCAO.

### Grid walking test

At 24 h before MCAO, rats were conditioned to cross the horizontal ladder. At 1, 2, 4, 6, and 10 days post-MCAO, each rat was placed on the grid and the number of foot-fault errors were monitored and recorded until the rats crossed the horizontal ladder.

### Sampling of serum and CSF

For sampling serum, rats were placed in a supine position, a needle was inserted into the heart, and blood samples were collected. Samples were centrifuged at 3000 rpm for 15 min and stored at −80 °C. For CSF sampling, rats were placed on a stereotaxic apparatus. The skin was incised, and a 27 G needle was inserted into the cisterna magna. When the tip of the needle was inserted 1–1.5 mm, reflux of the CSF was observed. Approximately 100 μl of CSF was withdrawn.

### Real-time PCR

RNA preparation and real-time PCR were performed as described previously^19^. Total RNA was purified using TRI reagent (Sigma, St. Louis, MO, USA; T9424), according to the manufacturer’s instructions. First-strand cDNA was synthesized using a Takara RNA PCR Kit (Doctor Protein, Seoul, Korea; DR01612) in a total volume of 20 μl containing 1 μg of total RNA. Real-time PCR was performed in a final volume of 20 μl containing 10 μl of 2× SYBR Green supermix (Takara Bio, Otsu, Japan; RR420), forward and reverse primers (1 μl each of 5 pmol/μl of both), and 5 μl of cDNA (50 ng; 1/100 dilution) using a Mini-Opticon Real-Time PCR System Detector (Bio-Rad, Richmond, CA, USA). PCR was performed as follows: 5 min at 95 °C, followed by 40 cycles of 30 s at 95 °C, 30 s at 57 °C, and 30 s at 72 °C. Specificity of amplification was determined by DNA melting-curve analysis using built-in software. Differences in amplification fold were calculated by real-time PCR amplification of the target gene using glyceraldehyde 3-phosphate dehydrogenase (GAPDH) as an internal standard using the built-in Gene Expression Analysis software in the iCycler iQ Real-Time RCR Detection System (Bio-Rad, Richmond, CA, USA). The following primer sets were used: 5′-CACCACGCTCTTCTGTCTACTG-3′ (forward) and 5′-GTACTTGGGCAGATTGACCTC-3′ (reverse) for TNF-α; 5′-GGAGAAGCTGTGGCAGCTA -3′ (forward) and 5′-GCTGATGTACCAGTTGGGGA-3′ (reverse) for IL-1β; 5′-GCTGTACAAGCAGTGGCAAA-3′ (forward) and 5′-GTCTGGAGTGGGAGGCACT-3′ (reverse) for cyclooxygenase-2; 5′-TCATTGACCTCAACTACATGGT-3′ (forward) and 5′-CTAAGCAGTTGGTGGTGCAG-3′ (reverse) for GAPDH; and 5′-GCATCCCAAGTACGAGTGGT-3′ (forward) and 5′-CCATGATGGTCACATTCTGC-3′ (reverse) for iNOS. Real-time PCR was performed in quadruplicate.

### Immunoblotting and co-immunoprecipitation analysis

Immunoblotting and co-immunoprecipitation studies were carried out as previously described^19^. Briefly, blood serum samples (30 μg) were prepared from animals in seven groups mentioned in a previous section (Sham, LPS, MCAO, MCAO+LPS, and MCAO+LPS/A box, MCAO+LPS/HPep1, MCAO+LPS+LPS-RS) and separated in 10% sodium dodecyl sulfate-polyacrylamide gels. After blocking membranes with 5% non-fat milk for 1 h, they were incubated with primary antibodies for anti-HMGB1 (1:2000; Abcam, Cambridge, UK; ab67281), anti-albumin (1:5000; Santa Cruz Biotechnology, Santa Cruz, CA, USA; SC-374670), and anti-α-tubulin (1:5000; Cell Signaling, Danvers, MA, USA; #2144) overnight at 4 °C. The next day, blots were detected using anti-rabbit or anti-mouse horse radish peroxidase-conjugated secondary antibody (1:2000; Santa Cruz Biotechnology; SC-2004 or SC-2005) and a Chemiluminescence Kit (Roche, Basel, Switzerland). Blood sera containing 500 μg of protein were immunoprecipitated with 2 μl of anti-LPS antibody (Abcam, Cambridge, UK; ab35654) overnight at 4℃. Pre-equilibrated protein A PLUS-Agarose beads (Pierce Biotechnology, Rockford, IL, USA; #20365) were then added and incubated for 2 h at 4 °C on a rotating wheel. Beads were washed three times with radioimmunoprecipitation assay buffer and separated by sodium dodecyl sulfate-polyacrylamide gel electrophoresis.

### Pull-down assay

Serum was incubated with biotin-IAM (1 μg/ml; Sigma Aldrich, St. Louis, MO, USA; B2059) for 18 h. Mixtures were incubated with 20 μl (50% slurry) of streptavidin-agarose beads (Pierce, Rockford, IL, USA; #20349) for 1 h at 4℃ with rotation and centrifuged at 8000 rpm for 1 min. The pellets contained reduced-HMGB1, while the supernatants contained disulfide-HMGB1. The supernatants were incubated with biotin-M (1 μg/ml; Sigma Aldrich, St. Louis, MO, USA; B1267) in the presence of dithiothreitol (10 mM) for 6 h and then with streptavidin beads for 1 h and then centrifuged at 8000 rpm. The pellets were washed three times and analyzed by immunoblotting. To show that HMGB1 binds to LPS, serum was preincubated with HMGB1 A box peptide (0.5 or 1 μg/ml; HMGbiotech, Milano, Italy; HM-012) for 6 h and then incubated with biotin-LPS (5 μg/ml; Invivogen, San Diego, CA, USA; tlrl-bblps) for 24 h. The mixture was precipitated using streptavidin-agarose beads for 1 h at 4℃ with rotation and analyzed by immunoblotting with anti-HMGB1 antibody (Abcam, Cambridge, UK; ab67281).

### Enzyme-linked immunosorbent assay

To detect IL-1β or TNF-α in blood serum, we used a solid-phase sandwich ELISA (enzyme-linked immunosorbent assay) Kit (eBioscience, San Diego, CA, USA; BMS630 or BMS622). Sera were prepared at the indicated times, and standard samples were diluted with distilled water and applied to ELISA plates. IL-1β or TNF-α concentrations were determined according to the manufacturer’s protocol. Absorbance levels were measured at 450 nm using an ELISA reader.

### Statistical analysis

Two-sample comparisons were performed using Student’s *t* test and multiple comparisons using one-way or two-way analysis of variance. Analysis was performed using PRISM software 5.0 (GraphPad Software). Results are presented as the means ± SEM and statistical difference was accepted at the 5% level.

## Electronic supplementary material

Supplemental Figure(DOCX 1239 kb)

Supplemental Table(DOCX 31 kb)
