# Supplementary material for: Alarmin HMGB1 induces systemic and brain inflammatory exacerbation in post-stroke infection rat model
Source: Cell Death Dis. 2018 Mar 19;9(4):426. doi: 10.1038/s41419-018-0438-8 (PMC5859283; doi:10.1038/s41419-018-0438-8)
Supplement: Supplementary file 1 — Supplemental Figure(DOCX 1239 kb) [file 41419_2018_438_MOESM1_ESM.docx]

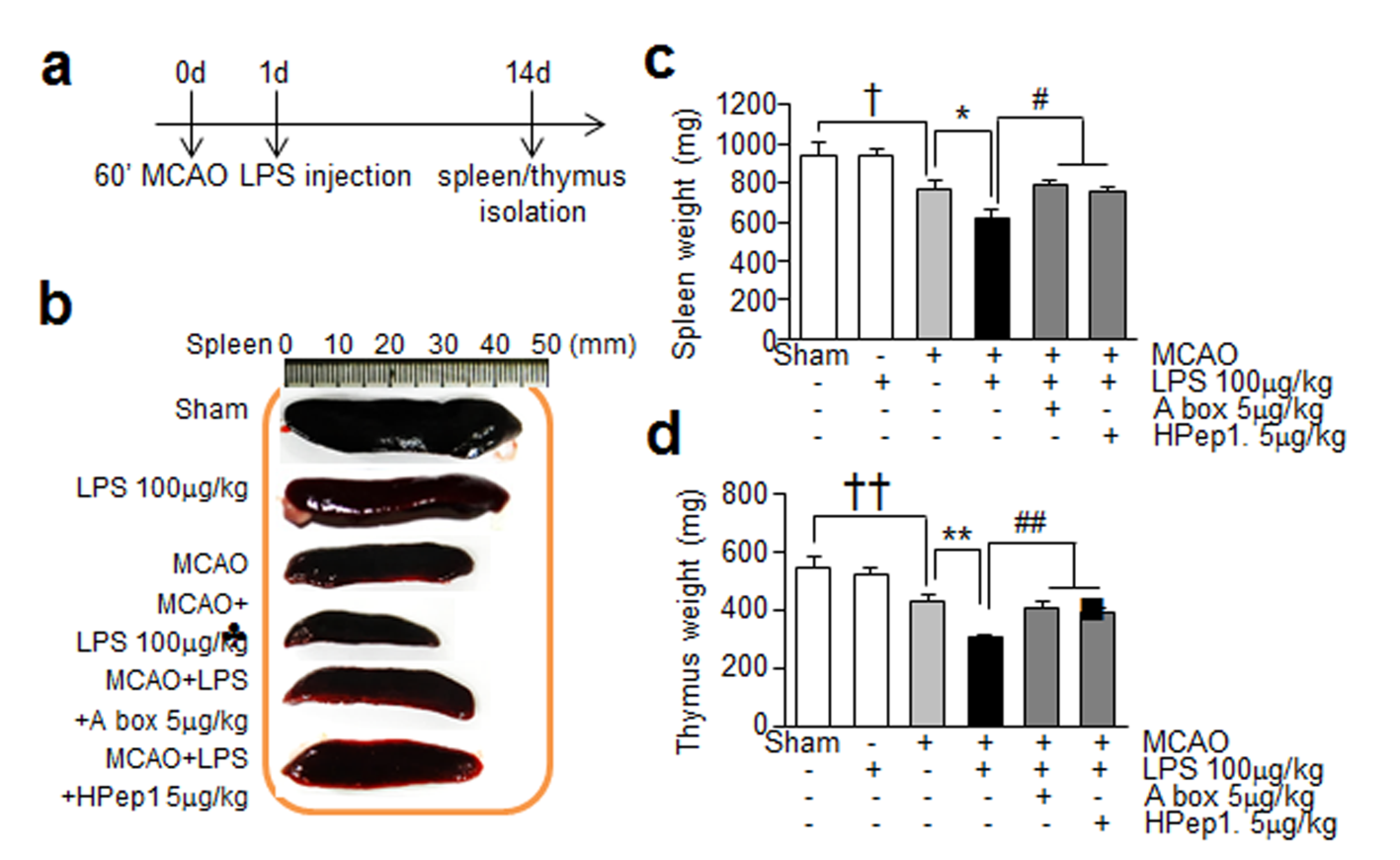


Supplementary Figure 1. Effects of systemic LPS injection on spleen and thymus in MCAO animals.

(a) LPS (100 μg/kg, i.p.) was administered at 24 hrs post-MCAO. At 14 days post-MCAO, spleen and thymus were removed and their weights and lengths were measured. Representative images of spleens (b) and mean weights of spleen (c) and thymus (d) are presented as means±SEMs (n=4-6). †p<0.05, ††p<0.01, *p<0.05, **p<0.01, #p<0.05, ##p<0.01 between the indicated groups.


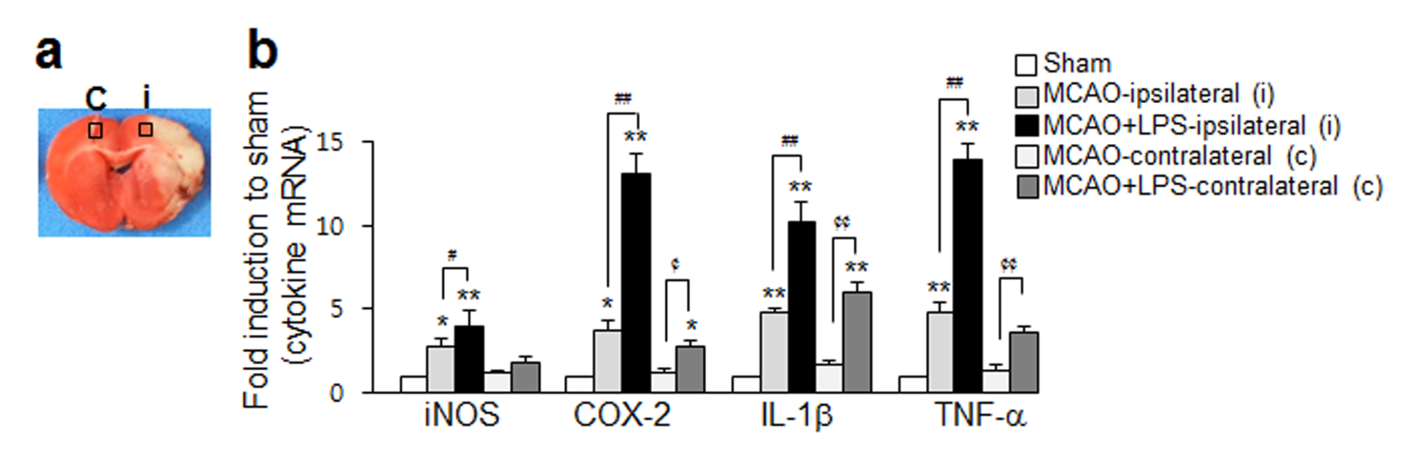


Supplementary Figure 2. Enhancement of brain inflammatory marker inductions in PSI animals

After injection of LPS (100 μg/kg, i.p.) at 24 hrs post-MCAO, levels of inflammatory markers were measured in cortex penumbra of the ipsilateral hemisphere (i) and contralateral side of the same area (c) of the MCAO and MCAO+LPS animals at 36 hrs post-MCAO by real-time PCR (a). Results are presented as means±SEMs (n=4) (b). Sham, sham-operated animal; MCAO-ipsilateral, ipsilateral side of treatment-naive MCAO animal; MCAO+LPS-ipsilateral, ipsilateral side of LPS-treated MCAO animal; MCAO-contralateral, contralateral side of treatment-naive MCAO animal; MCAO+LPS-contralateral, contralateral side of treatment-LPS+MCAO animal. *p<0.05, **p<0.01 versus sham controls, ^#^p<0.05, ^##^p<0.01 versus treatment-naive MCAO controls, ^¢^p<0.05, ^¢¢^p<0.01 versus treatment-naive MCAO-contralateral controls


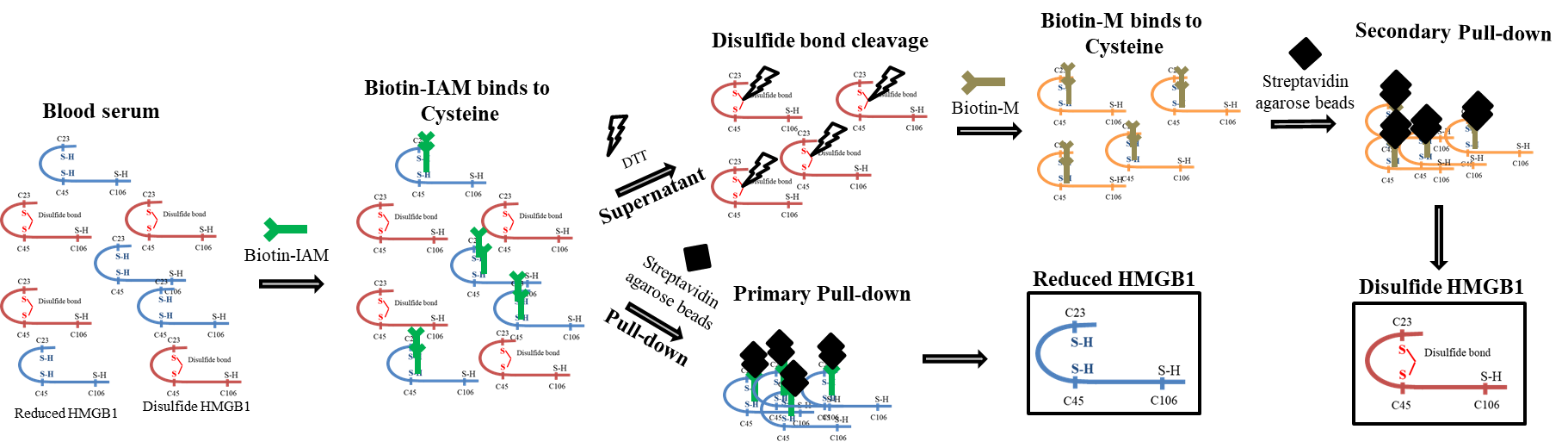


Supplementary Figure 3. Scheme of consecutive pull down assay

LPS (100 μg/kg, i.p.) was administered at 24 hrs post-MCAO and levels of disulfide- or reduced HMGB1 in sera in MCAO control or MCAO+LPS animals at 1 or 2 days post-MCAO were examined. Pull down assay for reduced HMGB1 was carried out using streptavidin agarose beads after incubating sera with biotinylated-Iodoacetamide (Biotin-IAM). Pull down assay for disulfide HMGB1 in supernatants was carried out after treating DTT and then incubating with biotinylated-maleimide (Biotin-M).


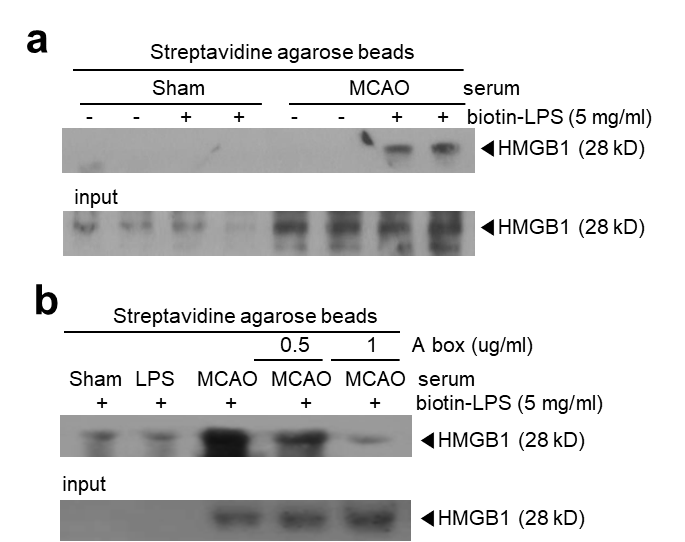


**Supplementary Figure 4. Binding activity of HMGB1 accumulated in sera of LPS-injected MCAO animals to LPS in *in vitro* pull-down assay**

(a) Serum was prepared from sham control and MCAO animals at 1 day post-MCAO and pull-down assays were performed using streptavidin agarose beads. Sera were incubated with biotinylated-LPS (5 μg/ml) for 6 hrs, and the amounts of HMGB1 interact with LPS were determined by immunoblotting. (b) Pull down assay was carried out in the presence of 0.5 or 1 μg /ml of HMGB1 A box. Amounts of HMGB1 before immunoprecipitations are presented as input controls and the results shown are representative of three independent experiments.


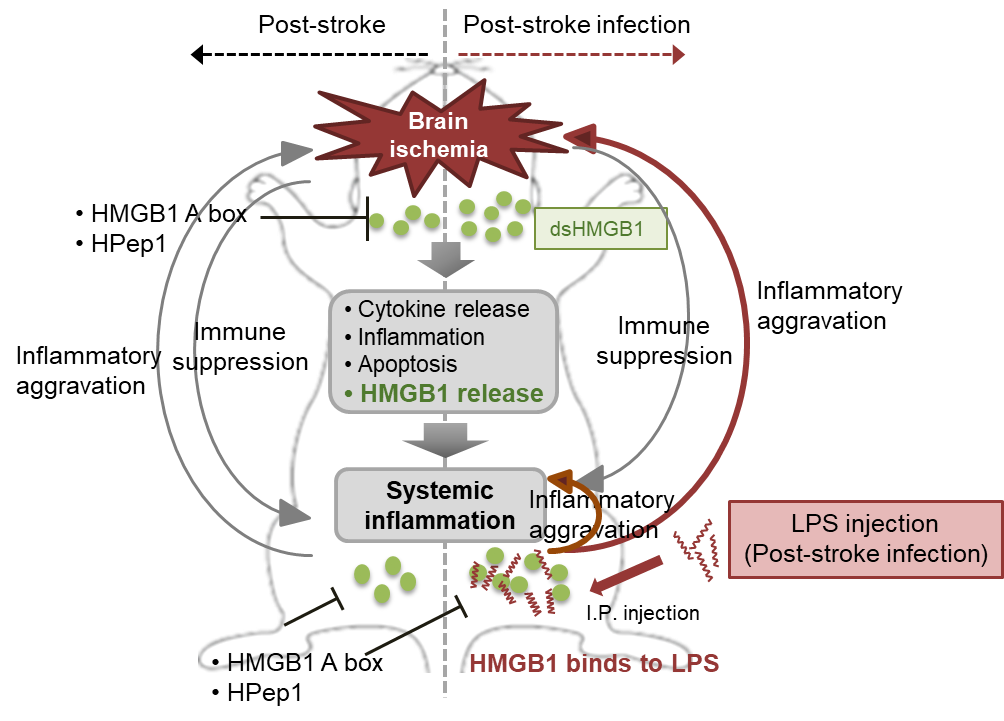


**Supplementary Figure 5. Aggravation of systemic and brain inflammation by HMGB1 in PSI.** Alarmin HMGB1 mediates potentiation of LPS function, exacerbating systemic and brain inflammation, expanding infarct volume, and worsening neurological and functional outcomes. There is a positive-feedback loop between augmentation of LPS function by HMGB1 and subsequent HMGB1 release/serum accumulation. HMGB1 binds to LPS and this interaction is inhibited by HMGB1 A box or Hpep1.
