# Supplementary material for: Alarmin HMGB1 induces systemic and brain inflammatory exacerbation in post-stroke infection rat model
Source: Cell Death Dis. 2018 Mar 19;9(4):426. doi: 10.1038/s41419-018-0438-8 (PMC5859283; doi:10.1038/s41419-018-0438-8)
Supplement: Supplementary file 2 — Supplemental Table(DOCX 31 kb) [file 41419_2018_438_MOESM2_ESM.docx]

**Table 1**

Control group MCAO group

Base LPS treat Base LPS treat LPS+A box treat

Table 1. Physiological parameters

Values are means±SD (n=4). One-way Analysis of variance revealed no significant intergroup difference for any variance.

Temperature, ℃ 37.4±0.4 37.3±0.1 37.2±0.2 37.1±0.2 37.2±0.2

pH 7.6±0.2 7.5±0.2 7.5±0.2 7.5±0.2 7.6±0.1

pO_2_, mmHg 150.2±9.0 152.1±15.8 150.7±3.2 147.3±4.6 143.4±10.3

pCO_2_, mmHg 37.6±1.8 39.5±2.4 43.5±4.1 42.3±7.4 43.4±5.5

Glucose, mg/dL 110.8±10.6 107.4±11.7 109.3±10.3 107.0±8.5 110.0±14.9
